# Supplementary material for: Inhibition of Mitochondrial Dynamics Preferentially Targets Pancreatic Cancer Cells with Enhanced Tumorigenic and Invasive Potential
Source: Cancers (Basel). 2021 Feb 9;13(4):698. doi: 10.3390/cancers13040698 (PMC7914708; doi:10.3390/cancers13040698)
Supplement: Supplementary file 1 [file cancers-13-00698-s001.pdf]

Figure S1, related to Figure 1

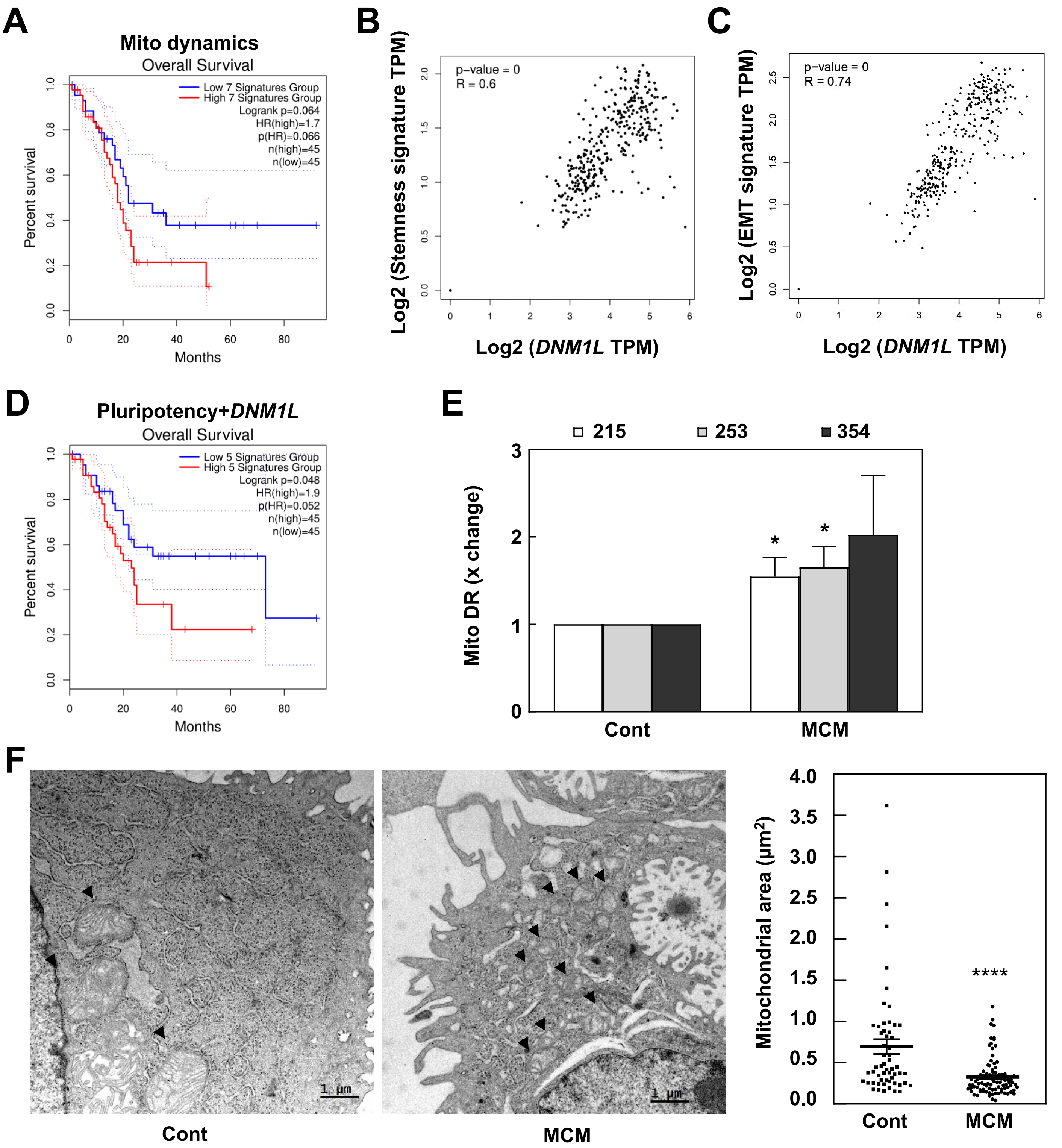

Figure S1. Mitochondrial fission relates to stemness and EMT in human PDAC. A. Overall survival of PDAC patients in the upper and lower quartiles for a mitochondrial dynamics signature (*DNM1L*, *DNM2*, *FIS1*, *MFF*, *MFN1*, *MFN2* and *OPA1*), showing Hazard Ratio (HR) value. The dotted blue and red lines depict the confidence intervals corresponding to the lower and higher quartile expression groups, respectively. **B**, **C**. Correlation of *DNM1L* expression and stemness (*NANOG*, *OCT4*, *KLF4*, *SOX2*) (B) or EMT signatures (*ZEB1*, *SNAI1* and *SNAI2*) (C). Calculated p-values are <1e-99. **D**. Overall survival of PDAC patients in the upper or lower quartiles for above stemness signature combined with *DNM1L* expression, showing HR value. The dotted blue and red lines depict the confidence interval corresponding to the lower and higher quartile expression groups, respectively. **E.** Mitochondrial mass as determined by flow cytometry using MitoTracker™ Deep Red FM (Mito DR) in either control cells or cells treated with conditioned media from M2-polarized macrophages (macrophage-conditioned media, MCM) (n=3-4). Control set as 1 for fold change. **F.** TEM images and quantification of the mitochondrial area of control cells *vs* cells treated with MCM (n=9-12 pictures representing 57 *vs* 94 mitochondria). $*p < 0.05$, $****p < 0.0001$ using the Mann & Whitney test.

Figure S2, related to Figure 2

A

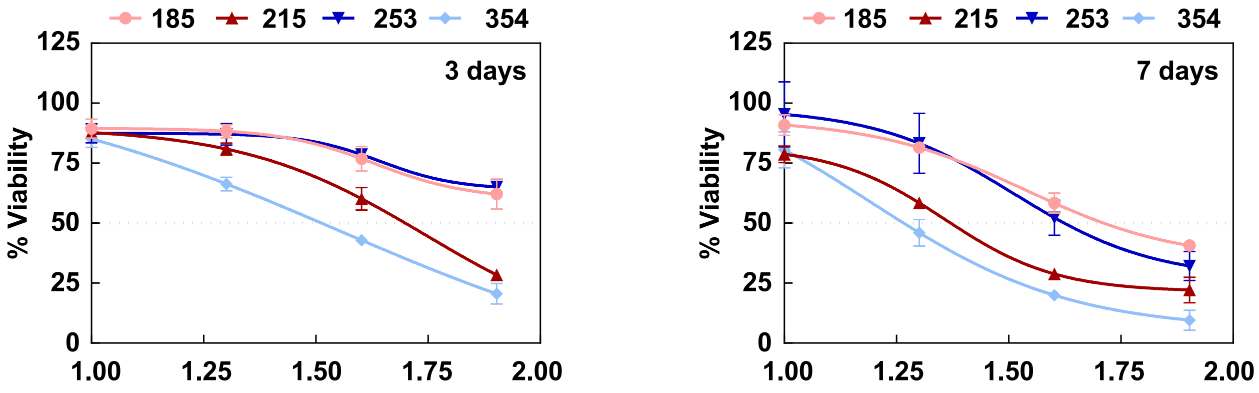

B

mDivi (µM)	EC50		IC50	
**PDXs**	**3d**	**7d**	**3d**	**7d**
**185**	n.a.	34.41	n.a.	54.23
**215**	55.57	22.74	52.77	22.99
**253**	n.a.	32.43	n.a.	42.43
**354**	35.44	15.58	32.51	18.74

C

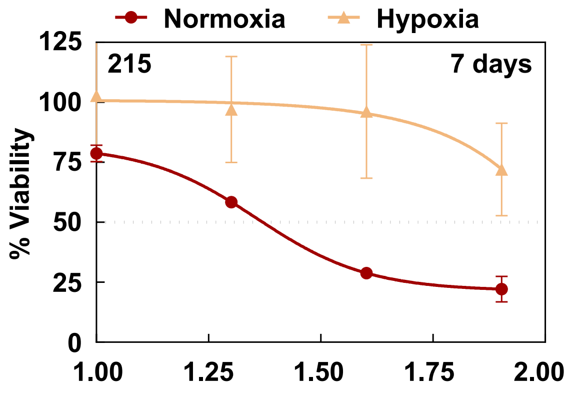

Figure S2. mDivi-1 decreases cell viability in normoxia. **A**, **B.** Evaluation of mDivi-1 EC50 for cell proliferation in normoxic condition (20% O_2) for 4 different PDX models after 3 and 7 days of treatment. In B, depicted EC50 and IC50 values wre obtained from the same dataset. n.a., not applicable. **C.** Evaluation of mDivi-1 EC50 for proliferation in normoxic or hypoxic (3% O_2) conditions for 215 cells after 7 days of treatment.

Figure S3, related to Figure 3

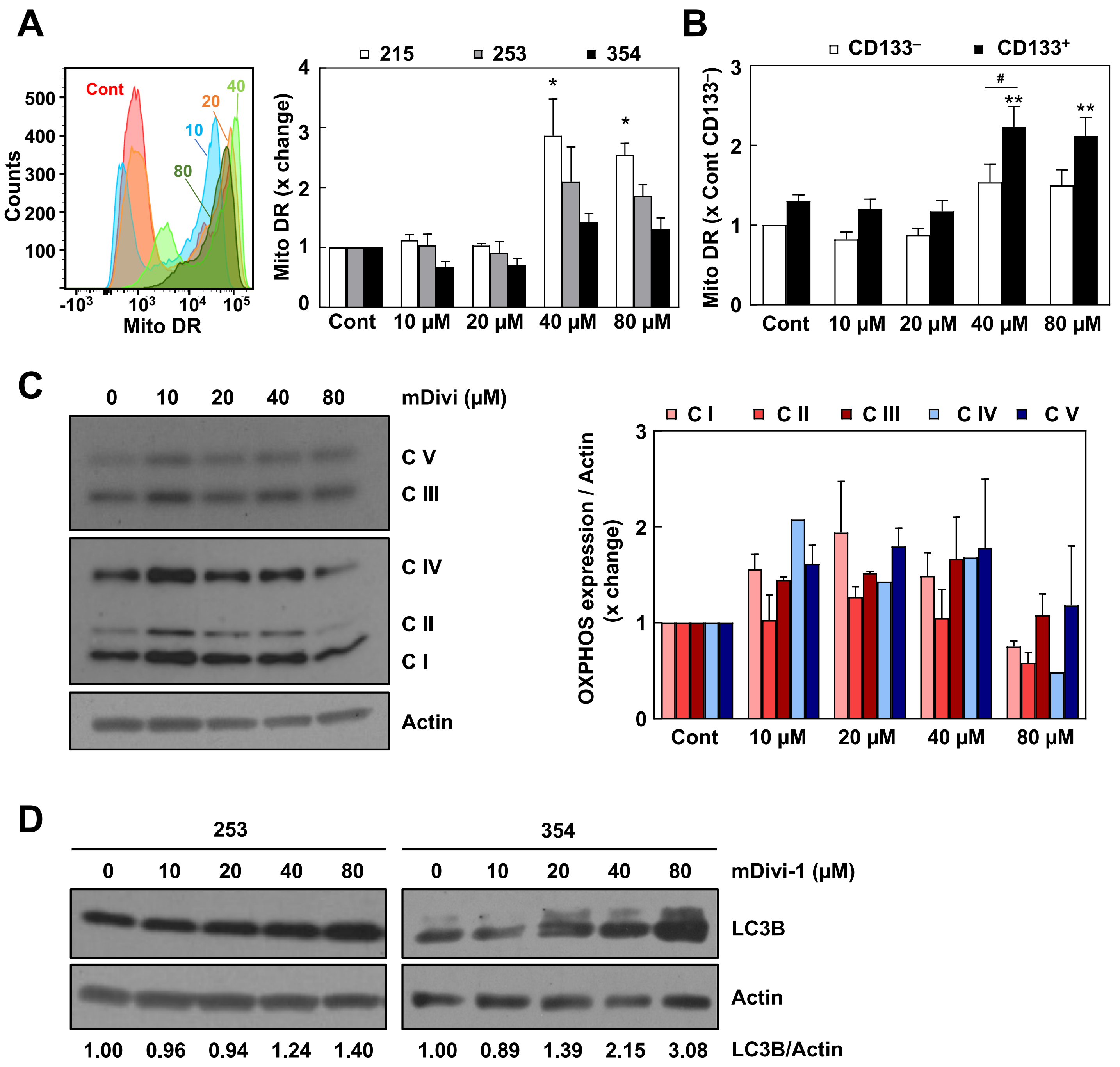

Figure S3. mDivi-1 disrupts mitochondrial function. A, **B.** Mitochondrial mass as measured by MitoTracker™ Deep Red FM (Mito DR) staining for the bulk cell population (A) and separated for $CD133^-$ *vs* $CD133^+$ cells (B) (n=4-9). Panel A on the left shows a representative plot Mito DR median staining for the bulk cell population. **C.** Protein expression for mitochondrial respiratory chain complexes as assessed by WB (left) and the corresponding densitometric quantification (right) for 354 cells (n=2, except for C IV with n=1). **D.** LC3B protein expression as assessed by WB for 253 and 354 cells. The numbers below indicate the quantification of expression relative to actin. In C, D, actin was used as loading control for densitometric analyses. * *vs* control condition, *p<0.05, **p < 0.01; # *vs* $CD133^-$ for the indicated condition, #p<0.05. Kruskal-Wallis with Dunn's post-test (A); ANOVA with Bonferroni *post-hoc* test (B). Control set as 1 for fold change.

Figure S4, related to Figure 4

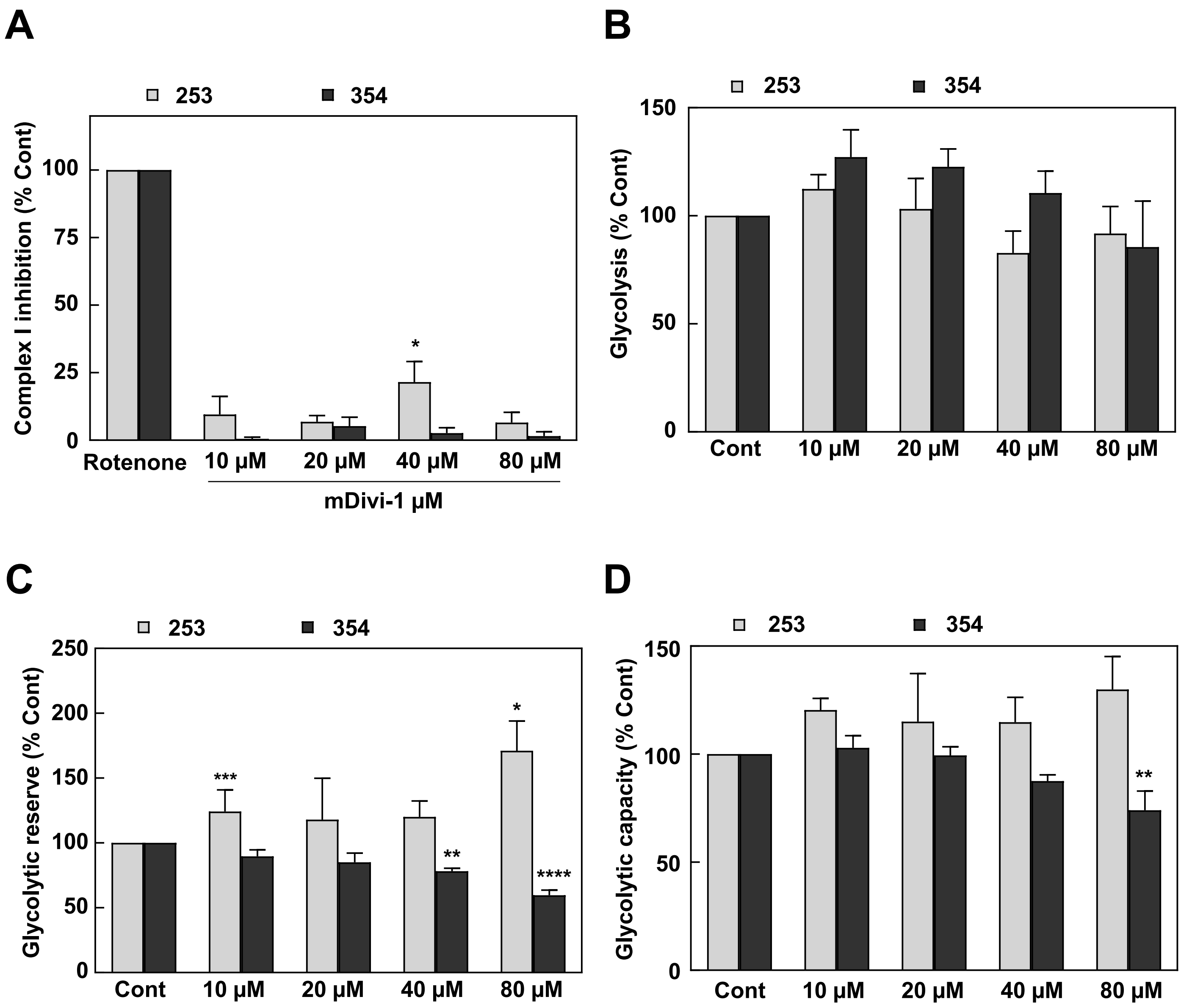

Figure S4. Treatment effects of mDivi-1 on cellular metabolism. A. Percentage of complex I inhibition following acute injection of mDivi-1 (n = 6) determined by the XF Extracellular Flux analyzer. Total complex I activity was determined based on basal OCR inhibition by the irreversible complex I inhibitor rotenone, used at 1 µM. Rotenone was injected after the last mDivi-1 measurement. **B**, **C**, **D.** Measurement of glycolytic rates using the XF Glycolysis Stress Test Kit in 253 and 354 cells after mDivi-1 treatment for 72h (n=3-6). Controls set as 100% for fold changes. *p<0.05, **p < 0.01, ***p < 0.001, ****p < 0.0001; Kruskal-Wallis with Dunn's post-test (A); ANOVA with Bonferroni *post-hoc* test (B-D).

Figure 1C

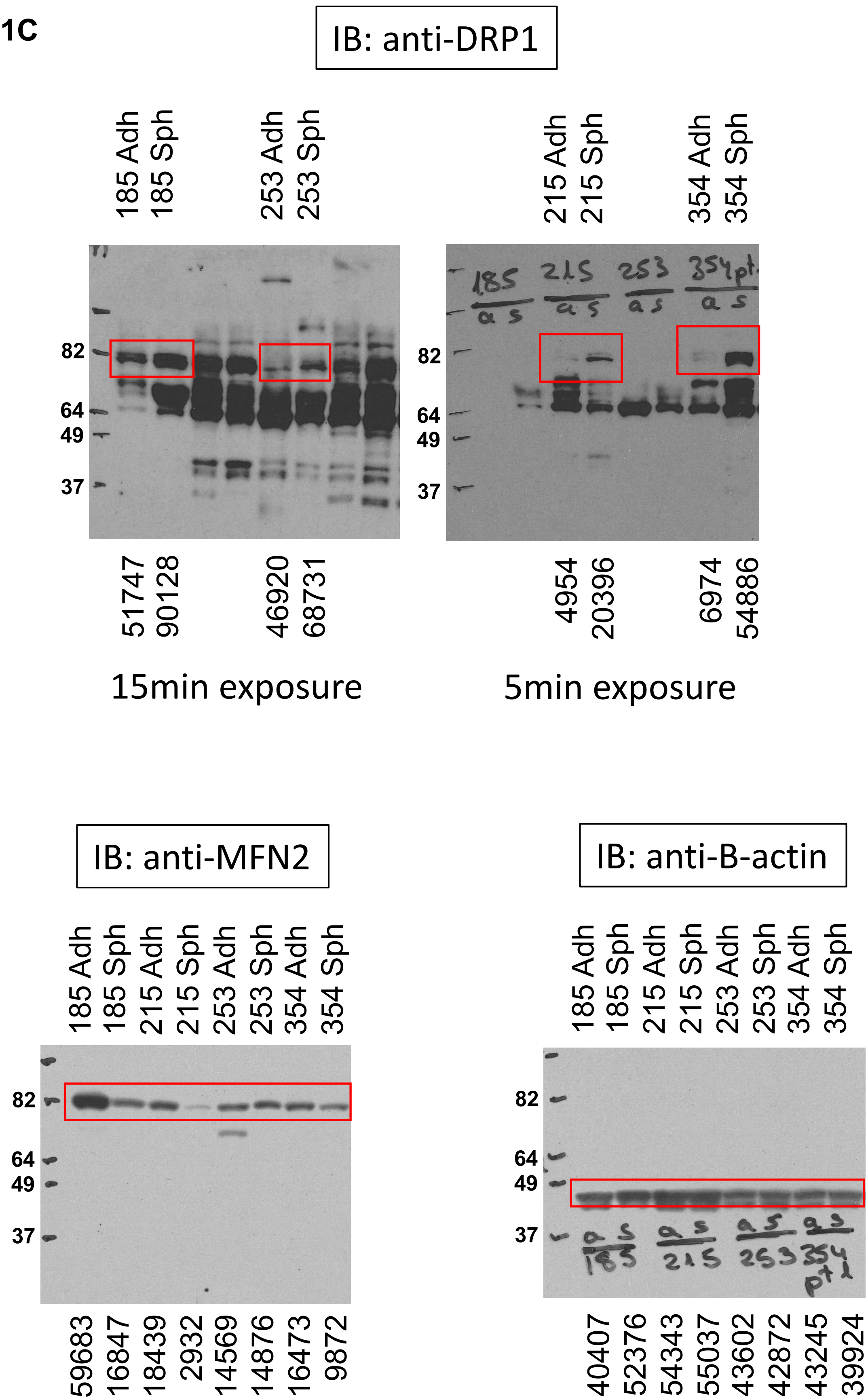
IB: anti-DRP1
185 Adh
185 Sph
253 Adh
253 Sph
215 Adh
215 Sph
354 Adh
354 Sph
82
64
49
37
51747
90128
46920
68731
4954
20396
6974
54886
15min exposure
5min exposure
IB: anti-MFN2
185 Adh
185 Sph
215 Adh
215 Sph
253 Adh
253 Sph
354 Adh
354 Sph
59683
16847
18439
2932
14569
14876
16473
9872
IB: anti-B-actin
40407
52376
54343
55037
43602
42872
43245
39924

Figure 1C

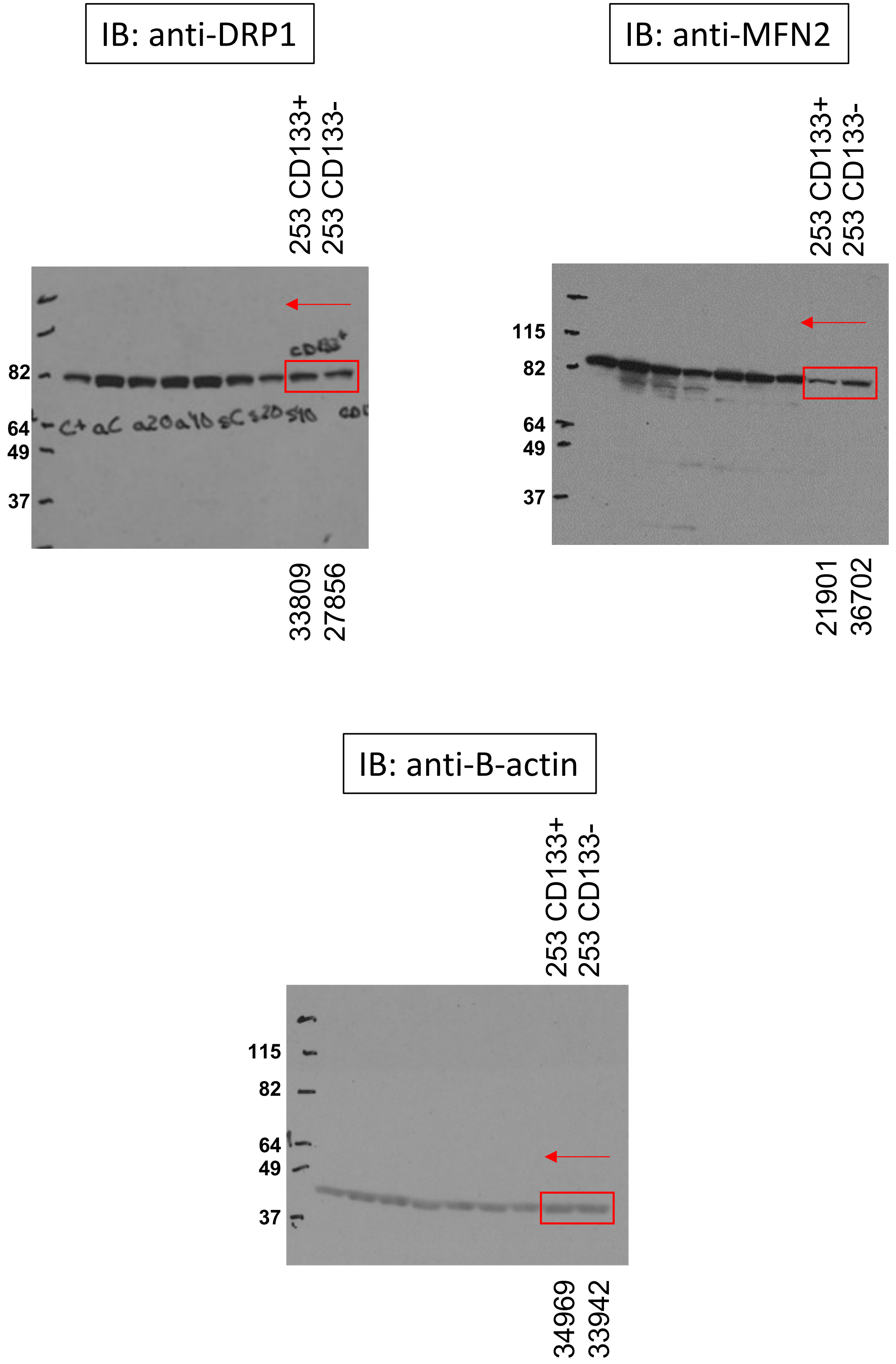
IB: anti-DRP1
253 CD133+
253 CD133-
82
64
49
37
33809
27856
IB: anti-MFN2
253 CD133+
253 CD133-
115
82
64
49
37
21901
36702
IB: anti-B-actin
253 CD133+
253 CD133-
115
82
64
49
37
34969
33942

Figure S3C

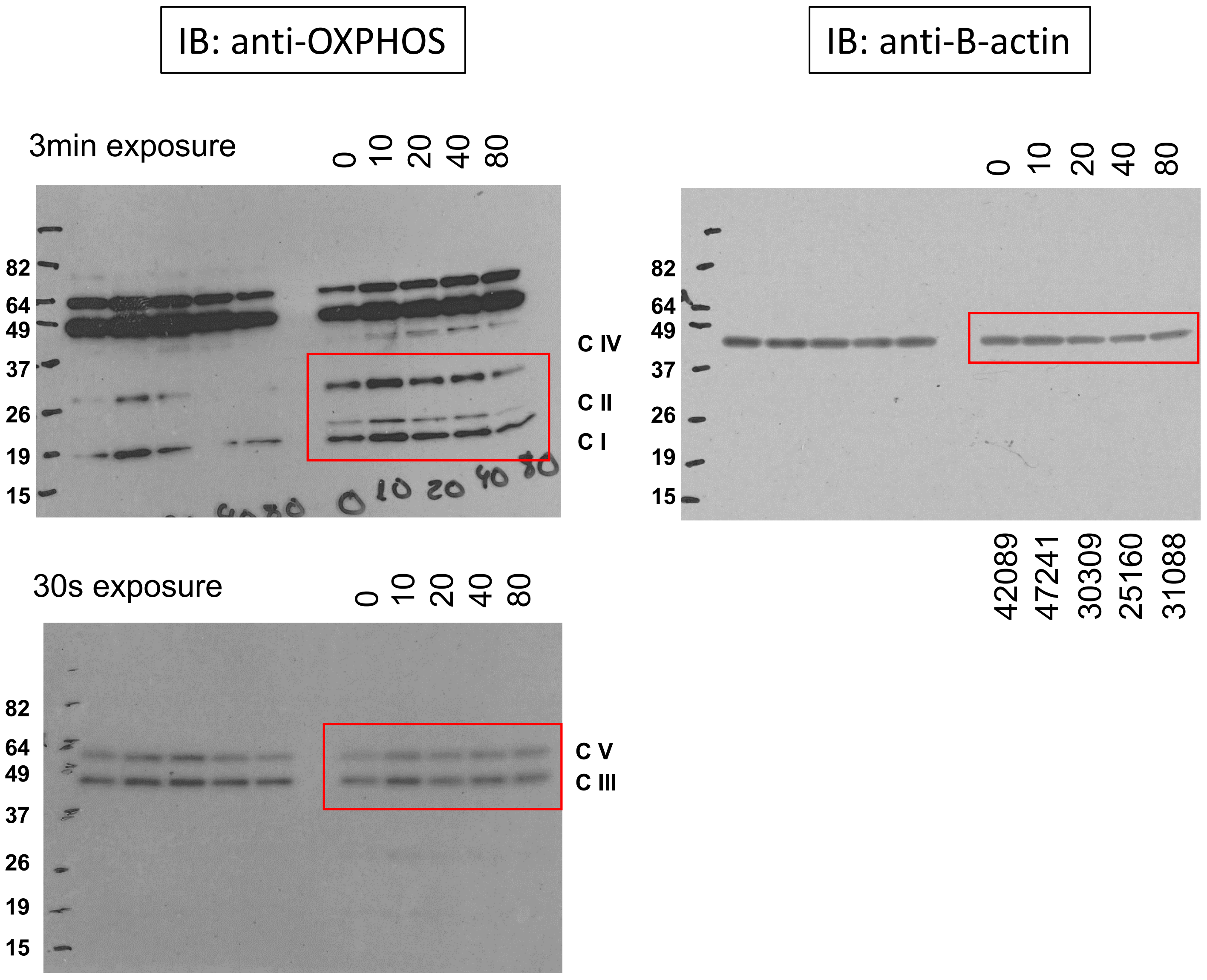

mDivi-1 µM	0	10	20	40	80
C IV	17860	41634	18394	17952	6390
C II	29510	42783	24646	23805	10414
C I	27515	43331	27899	28422	16467
C V	20423	41462	29211	30461	27171
C III	24961	41328	26856	31359	24004

Figure S3D

IB: anti-LC3B

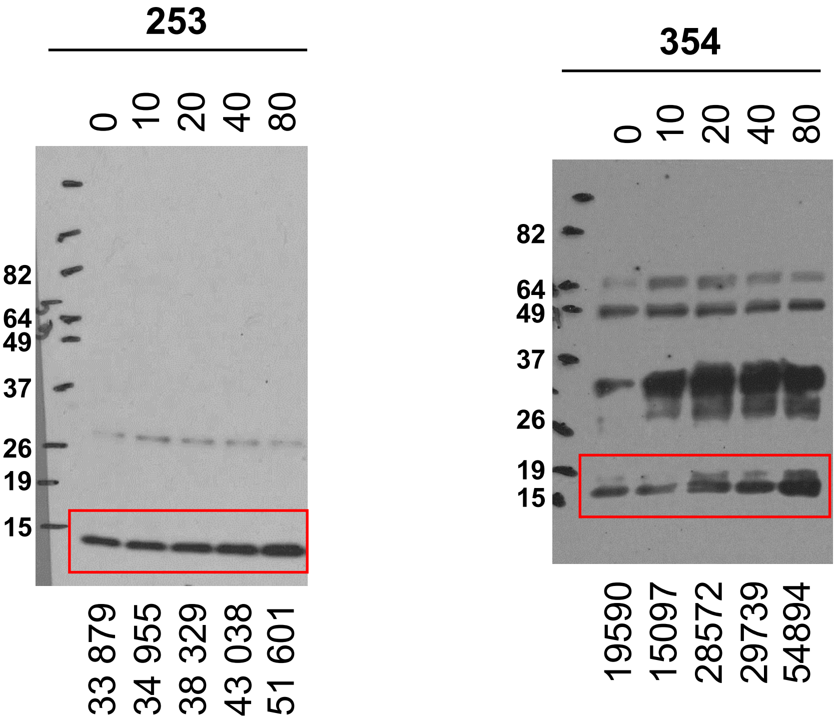

IB: anti-B-actin

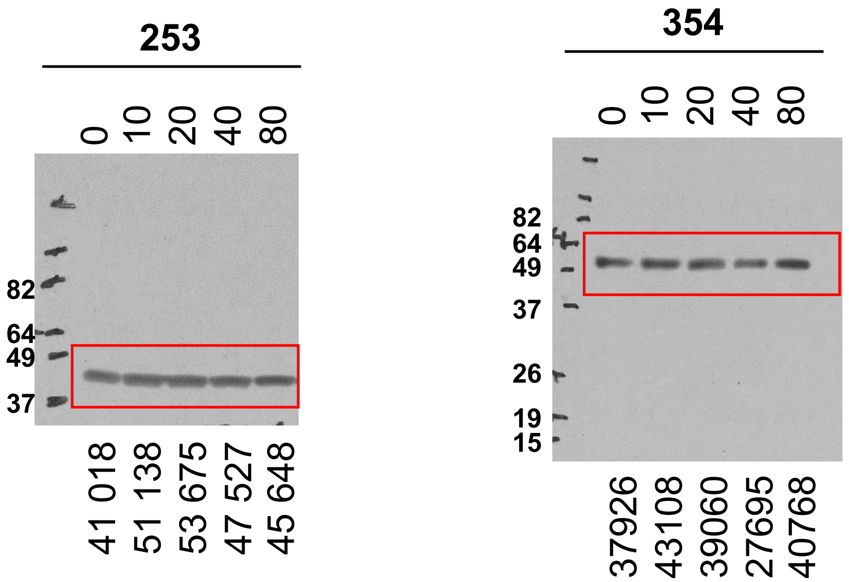

Figure 4E

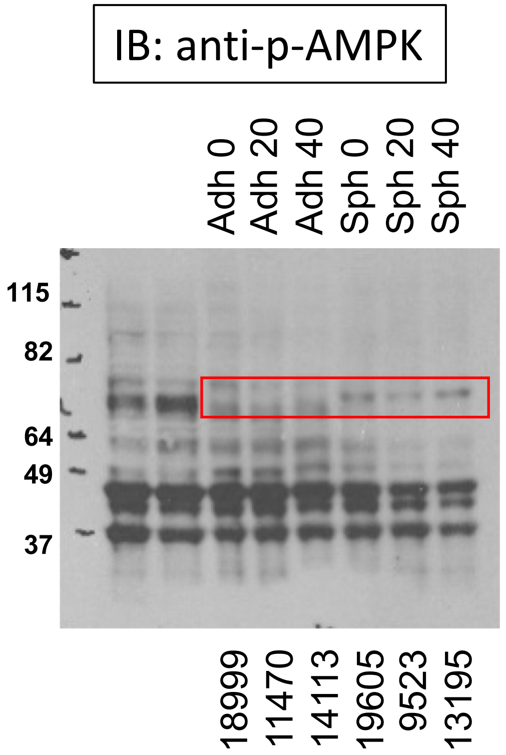
IB: anti-p-AMPK
Adh 0
Adh 20
Adh 40
Sph 0
Sph 20
Sph 40
115
82
64
49
37
18999
11470
14113
19605
9523
13195

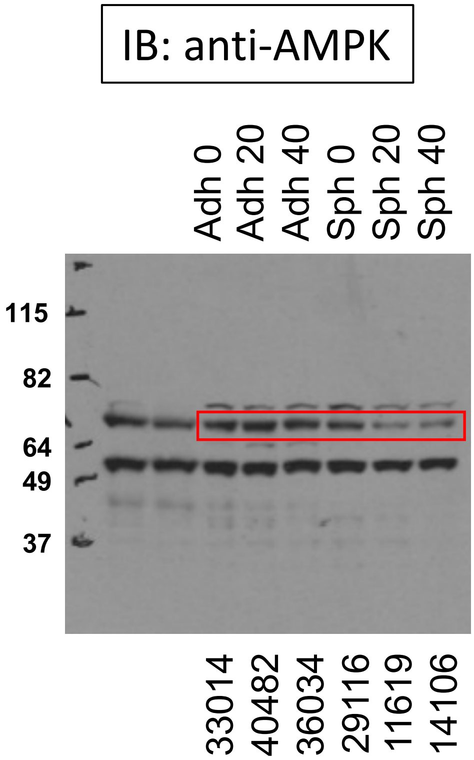
IB: anti-AMPK
Adh 0
Adh 20
Adh 40
Sph 0
Sph 20
Sph 40
115
82
64
49
37
33014
40482
36034
29116
11619
14106

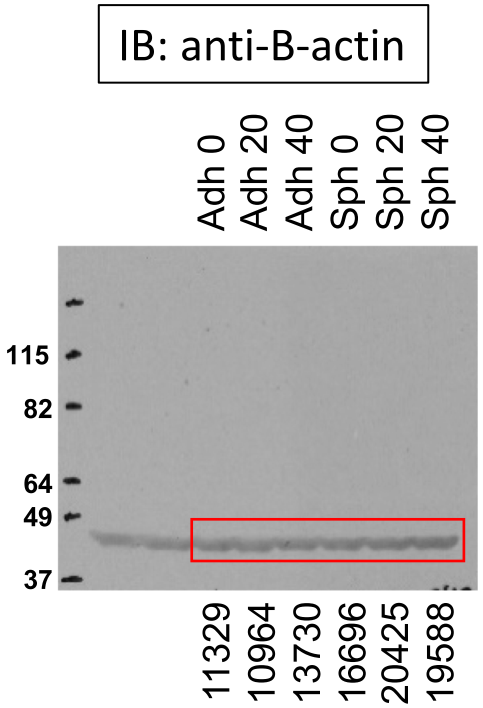
IB: anti-B-actin
Adh 0
Adh 20
Adh 40
Sph 0
Sph 20
Sph 40
115
82
64
49
37
11329
10964
13730
16696
20425
19588
